# Nitrous oxide, methane emissions and grain yield in rainfed wheat grown under nitrogen enriched biochar and straw in a semiarid environment

**DOI:** 10.7717/peerj.11937

**Published:** 2021-08-19

**Authors:** Stephen Yeboah, Wu Jun, Cai Liqun, Patricia Oteng-Darko, Erasmus Narteh Tetteh, Zhang Renzhi

**Affiliations:** 1 CSIR-Crops Research Institute, Kumasi, Ghana; 2 College of Resources and Environmental Sciences, Gansu Agricultural University, Gansu, China; 3 Gansu Provincial Key Lab of Arid Land Crop Science, Gansu Agricultural University, Gansu, China; 4 Kwame Nkrumah University of Science and Technology (KNUST), Kumasi, Ghana

**Keywords:** Semi-arid, Greenhouse gas, Rain-fed, Loess plateau, Grain yield, Carbon amendments, Fertilization

## Abstract

**Background:**

Soil application of biochar and straw alone or their combinations with nitrogen (N) fertilizer are becoming increasingly common, but little is known about their agronomic and environmental performance in semiarid environments. This study was conducted to investigate the effect(s) of these amendments on soil properties, nitrous oxide (N_2_O) and methane (CH_4_) emissions and grain and biomass yield of spring wheat (*Triticum aestivum* L.), and to produce background dataset that may be used to inform nutrient management guidelines for semiarid environments.

**Methods:**

The experiment involved the application of biochar, straw or urea (46% nitrogen [N]) alone or their combinations. The treatments were: CN_0_–control (zero-amendment), CN_50_ –50 kg ha^–1^ N, CN_100_–100 kg ha^–1^ N, BN_0_ –15 t ha^–1^ biochar, BN_50_–15 t ha^–1^ biochar + 50 kg ha^–1^ N, BN_100_–15 t ha^–1^ biochar + 100 kg ha^–1^ N, SN_0_ –4.5 t ha^–1^ straw, SN_50_ –4.5 t ha^–1^ straw + 50 kg ha^–1^ N and SN_100_–4.5 t ha^–1^ straw + 100 kg ha^–1^ N. Fluxes of N_2_O, CH_4_ and grain yield were monitored over three consecutive cropping seasons between 2014 and 2016 using the static chamber-gas chromatography method.

**Results:**

On average, BN_100_reported the highest grain yield (2054 kg ha^–1^), which was between 25.04% and 38.34% higher than all other treatments. In addition, biomass yield was much higher under biochar treated plots relative to the other treatments. These findings are supported by the increased in soil organic C by 17.14% and 21.65% in biochar amended soils (at 0–10 cm) compared to straw treated soils and soils without carbon respectively. The BN_100_treatment also improved bulk density and hydraulic properties (*P* < 0.05), which supported the above results. The greatest N_2_O emissions and CH_4_ sink were recorded under the highest rate of N fertilization (100 kg N ha^–1^). Cumulative N_2_O emissions were 39.02% and 48.23% lower in BN_100_ compared with CN_0_ and CN_100_, respectively. There was also a ≈ 37.53% reduction in CH_4_ uptake under BN_100_compared with CN_0_–control and CN_50_. The mean cumulative N_2_O emission from biochar treated soils had a significant decrease of 10.93% and 38.61% compared to straw treated soils and soils without carbon treatment, respectively. However, differences between mean cumulative N_2_O emission between straw treated soils and soils without carbon were not significant. These results indicate the dependency of crop yield, N_2_O and CH_4_ emissions on soil quality and imply that crop productivity could be increased without compromising on environmental quality when biochar is applied in combination with N-fertilizer. The practice of applying biochar with N fertilizer at 100 kg ha^−1^ N resulted in increases in crop productivity and reduced N_2_O and CH_4_soil emissions under dryland cropping systems.

## Introduction

Atmospheric methane (CH_4_) and nitrous oxide (N_2_O) are persistent greenhouse gases (GHG) influencing global warming (IPCC, 2014). Agriculture contributes significant amounts of N_2_O and CH_4_ to the atmosphere, however net GHG emissions as CO_2_ from farming-related activities can be potentially reduced by increasing carbon (C) sequestration in soil and crop biomass (Wang et al., 2021). This may be achieved by implementing improved crop and fertilizer management practices that maximize biomass production and C returned to soil (Norton, 2014). There are no significant terrestrial sinks of N_2_O hence reduction in its emission may only be achieved by managing nitrogen (N) inputs, and improving soil conditions and efficiency of applied fertilizer-N (Grace, 2016). However, in semi-arid regions of China, in an attempt to increase yields, farmers are compelled to apply more fertilizer, leading to an over-application (Xu & Yang, 2017). There is heavy dependence on mineral fertilizers to ensure adequate N supply for crops, and in most cases more fertilizer is applied than needed by the plant (Liu et al., 2016). This is a common practice in most farming communities in semi-arid regions of China (Wang et al., 2021). The situation has led to negative impact on the environment, and threatens the long-term sustainability of Chinese agriculture (Liu et al., 2016; Wang et al., 2021). Therefore, it is key to identify suitable agricultural practices that could help maximize crop production without compromising on environmental quality.

Current increases in atmospheric GHG levels require that novel approaches are undertaken to mitigate impacts of climate change, such as management practices capable of improving soil C sequestration (Woolf et al., 2010). Soil carbon sequestration through application of recalcitrant C-rich biochar is mentioned as a suitable means to mitigate climate change, and improve soil fertility (Laird et al., 2010) and crop productivity (Steiner et al., 2007). According to Saggar (2010) N_2_O emissions are driven by the applications of fertilizer nitrogen (N), soil tillage and crop type, with their effects dependent on soil and weather conditions. Biochar application as a soil amendment, could therefore be an effective strategy for mitigating emissions and increasing crop yield. However, the effect of biochar on soil properties, GHG emissions and crop yield have been diverse. Several mechanisms have also been proposed in literature to explain the diverse effects, with limited amounts of evidence to support them. Yanai, Toyota & Okazaki (2007) reported decreased N_2_O and CH_4_ soil emissions in response to biochar application. In contrast, Clough et al. (2013) observed no suppression of N_2_O and CH_4_ soil emissions, whilst similar effect was observed by Zhang et al. (2010). Zhang et al. (2011) also reported that biochar application in dryland significantly reduces soil CH_4_ emission by 33% compared to soil without biochar. Zimmerman, Gao & Ahn (2011) attributed the positive effect of biochar application on soil CH_4_ emissions to the inhibition of soil methanotrophs while Zhu et al. (2018) associated reduced soil CH_4_ emissions to the change in the ratio of methanogenic to methanotrophic archaea. In general, most studies have found biochar amendments to either decrease or not significantly affect soil N_2_O emissions; however, some few reports have found increased N_2_O emissions following biochar amendments (Yeboah et al., 2018). Explanations for continued long-term suppression of N_2_O emissions in biochar-amended soils include alterations in microbial communities due to physical habitat changes, physical and/or chemical protection of organic C and/or N by biochar and alteration of micro-scale soil redox status due to electrochemical properties of biochars (Rivka, David & Timothy, 2019). It is thus clear that, these effects have been shown to vary significantly depending upon the type of biochar used and the environmental and soil conditions under which the material is applied.

The Loess Plateau is an important agricultural area in China and is widely used for grain production (He et al., 2014). The area is one of the most severely eroded regions in China, which coupled with limited precipitation and high evaporation rates, often results in poor crop productivity (He et al., 2014). Many studies have indicated that human activities, such as land use is responsible for the degradation and loss of soil fertility in semi-arid regions of China (Xu & Yang, 2017; Zhang et al., 2017; Huang et al., 2019). Traditional methods of soil cultivation often accelerates the decline of soil fertility, and loss of soil organic C (Lamptey, Li & Xie, 2018). Given the fact that the population of semi-arid regions in China mainly relies on rainfed agriculture for their livelihood; developing environmentally friendly and sustainable nutrient management strategies is crucial. There is limited information on the specific impact of widely-used agronomic practices involving biochar, straw and nitrogen fertilizer used alone or combined on greenhouse gas emission and crop yield in drier lossiah soils (Solomon et al., 2007). Moreover, little is known about the effect of biochar application to soil under arid conditions (Arfaoui, Ibrahimi & Trabelsi, 2019). This study hypothesized that increased C inputs would raise the soils potential to reduce N_2_O and CH_4_ soil emissions whilst increasing grain yield. Therefore, the objectives of this study were to: 1) determine the effect of biochar, straw and nitrogen fertilizer applied alone or combined with fertilizer-N on soil properties, (2) assess the effect of biochar, straw and nitrogen fertilizer applied alone or combined with fertilizer-N on biomass and grain yield of spring wheat, and (3) determine the effects of biochar, straw and nitrogen fertilizer used alone or combined with fertilizer-N on N_2_O and CH_4_ emissions.

## Materials & Methods

### Study site

**Figure 1:**
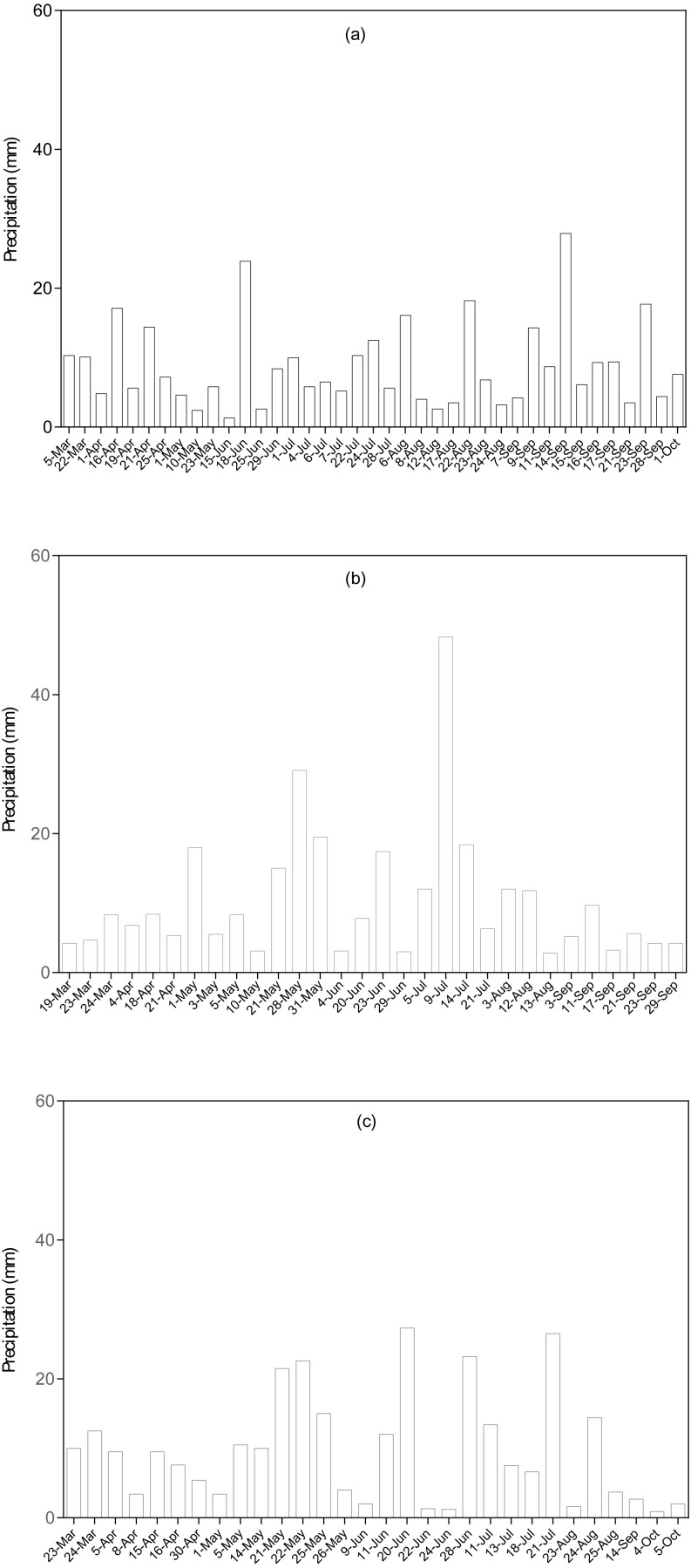
Precipitation (mm) in (A) 2014, (B) 2015 and (C) 2016 cropping season at the experimental site.

The study was conducted during the 2014, 2015 and 2016 growing seasons at the Dingxi Experimental Station (35°28′N, 104°44′E, elevation 1971-m above-sea-level) of the Gansu Agricultural University in Northwestern China. The research station is located in the semiarid Western Loess Plateau, which is characterized by step hills and deeply eroded gullies (Feng et al., 2013). This area has Aeolian soils, locally known as Huangmian (Chinese Soil Taxonomy Cooperative Research Group, 1995), which equate to Calcaric Cambisols based on the FAO (1990) description. The soil type in the study area is sandy-loam with low fertility. The soil has a pH of ≈8.3, soil organic carbon (SOC) ≤8.13 g kg^−1^, and Olsen-P ≤13 mg kg^−1^ as described in Yeboah et al. (2018). The type of soil in the study area is the principal soil for cultivation of crops in the agro-ecological zone. Long term average rainfall, evaporation and aridity in the study area is 391.9 mm per annum; 1531 mm per annum and 2.53 respectively. The aridity index (AI) is the degree of dryness of the climate at the study area. In July, the daily maximum temperature can increase to 38 °C. Similarly, in January daily minimum temperature can drop to −22 °C. Annual cumulative temperatures >10 °C are 2240 °C and annual radiation is 5930 MJ m^−2^, with 2477 h of sunshine as described in Yeboah et al. (2018). The agro-climatic conditions are similar to semiarid environments. The research site is characterized by continuous cultivation of the same field using conventional tillage practices. The preceding crop cultivated at the research site was potatoes (*Solanum tuberosum* L.). Seasonal rainfall recorded in 2014, 2015 and 2016 during the research was 174.6, 252.5 and 239.4 mm respectively (Fig. 1).

### Experimental design and description of treatment

The experiment involved addition of different carbon (C) sources; namely: biochar and straw, and N fertilizer in the form of urea (46% N) arranged in a randomized block design with nine treatments and three replications (Yeboah et al., 2018). The treatments were: CN_0_–control (zero-amendment), CN_50_ –50 kg ha^−1^ N applied each year, CN_100_ –100 kg ha^−1^ N applied each year, BN_0_ –15 t ha^−1^ biochar applied in a single dressing in 2014, BN_50_ –15 t ha^−1^ biochar applied in a single dressing in 2014 + 50 kg ha^−1^ N applied each year, BN_100_ –15 t ha^−1^ biochar applied in single dressing in 2014 + 100 kg ha^−1^ N applied each year, SN_0_ –4.5 t ha^−1^ straw applied each year, SN_50_ –4.5 t ha^−1^ straw applied each year + 50 kg ha^−1^ N applied each year and SN_100_ –4.5 t ha^−1^ straw applied each year + 100 kg ha^−1^ N applied each year as described in Yeboah et al. (2018). The two C sources (biochar and straw) were applied at the same quantity based on the straw returned to the soil every year and straw C mineralization. Biochar was spread evenly on the soil surface in March 2014 and incorporated into the soil using a rotary tillage implement to a depth of ≈10 cm. The biochar was obtained from Golden Future Agriculture Technology Company Limited, Liaoning in China. Biochar was produced from maize straw using pyrolysis process at a temperature of 350–550 °C. This process converted about 35% of the maize straw to biochar. The biochar in the form of granules was milled to a size of <5 mm to allow for even mixing with the soil. The wheat crop of the previous season from the research station was used as a source of straw for the study. In the plots that received straw treatment, the straw from the previous wheat crop was weighed and returned to the original plots. This was done after threshing. Biochar analysis was conducted using the procedure as describe in Lu (2000). Total C and N and soil pH were determined using a CN Analyzer (analytikjena; multi N/C, 2100S, Germany) and Kjeldahl digestion and distillation (Bremner & Mulvaney, 1982) and pH meter (model: Sartorius PB–10, Germany). The soil pH was determined using soil to water ratio of 1: 2.5. Similar protocol was used to determined total C and N, ash content and pH of the straw. Table 1 shows the chemical characterization of biochar and straw used in the experiment. All the treatments received a blanket application of Phosphorus (P) fertilizer which was applied equally at a rate of 46 kg ha^−1^ P in the form of ammonium dihydrogen phosphate (12% N, 52% P_2_O_5_). No–tillage seeder was used to incorporate the fertilizer to about 20 cm soil depth at planting. Based on the protocol described in Yeboah et al. (2016) Spring wheat (*Triticum aestivum* L. cv. Dingxi 35) was sown in Mid-March at a rate of 188 kg ha^−1^ seeds at 20-cm row spacing. The crop was harvested either at the end of July or early August. The individual plot’s measured 3 m by 6 m and the plots were separated by 0.5 m width protection rows.

**Table 1:** Characterization of biochar and straw used in the study.

				%					
Parameter	pH	BD (g cm^−3^)	SA (m^2^ g^−1^)	Ca	Mg	K	C	N	P	Ash content (%)
Biochar	9.2	0.68	8.75	0.8	0.47	0.51	53.28	1.04	0.26	25.5
Straw	6.5	/	/	0.53	0.04	0.47	45.05	0.94	0.08	8.9

**Notes.**

Values are means for *n* = 2.

### Soil sampling, measurements and analyses

Based on the protocol described in Yeboah et al. (2016), soil bulk density (BD) was determined by taking small cores and relating the oven–dried mass of soil to the volume of the core. Soil saturated hydraulic conductivity (Ksat) was determined at two points per plot using the disc permeameter method according to Carter (1993). Soil samples were collected from 0–10 and 10–30 cm depth and bulked for analysis. The samples were processed for analysis using the protocol described in Yeboah et al. (2018). Soil organic carbon (SOC) in the fine ground samples was determined by the modified Walkley & Black (1934) wet oxidation method (Nelson & Sommers, 1982).

### Gas sampling and analysis

Collection of N_2_O and CH_4_ gases were performed using the static chamber technique based on the procedure described by Zou et al. (2005). For each sampling event, gas collection was consistently performed between 08:00–12:00 h, based on the guidelines of Yeboah et al. (2016). Collection of samples for N_2_O and CH_4_ analyses was conducted at 0, 10, and 20 min after chamber closure. Samples were collected between March and September and detailed sampling procedure could be found in Yeboah et al. (2016); Yeboah et al. (2018). Based on earlier studies conducted in low rainfall areas (*e.g.*, Wang et al., 2010) emissions occurring during the dry season were expected to be low and therefore did not justify measurements over that period. Gas fluxes were measured over 14 sampling events per year. Whilst acknowledging that accurate estimates of total emissions cannot be determined from relatively few sampling events, the main purpose of this work was to quantify relative differences between-treatments, which therefore justifies the approach used in this study. A similar approach was also employed by Tullberg et al. (2018) to quantify soil emissions of GHG from tillage and traffic treatments in conservation agriculture areas with seasonal rainfall. The N_2_O and CH_4_ concentration in samples were analyzed within 2 to 3 days after collection using gas chromatograph (GC). The GC system (Agilent 7890A, USA) equipped with flame ionization detector (FID) was used for CH_4_ analysis and an electron capture detector (ECD) was used for N_2_O analysis. Rates of CH_4_ and N_2_O fluxes were calculated by linear increment of the gas concentration at 0, 10 and 20 min. The calculation was only accepted when the R^2^ of the linear correlation was higher than 0.90 (*p* < 0.05). The average GHG fluxes were a mean of three replicates of each treatment over the sampling dates. Further procedure for the analysis and conditions of the column could be found in Yeboah et al. (2018) and Zou et al. (2005).

### Estimations of nitrous oxide and methane emissions

The N_2_O (mg m^−2^ h^−1^) and CH_4_ (mg m^−2^ h^−1^) emissions were calculated using Eq. (1) based on the protocol described in Yeboah et al. (2016):

(1) \begin{eqnarray*}F= \frac{{C}_{2}\times V\times {M}_{0}\times 273/{T}_{2}-{C}_{1}\times V\times {M}_{0}\times 273/{T}_{1}}{A\times \left( {t}_{2}-{t}_{1} \right) \times 22.4} \end{eqnarray*}

where: F are fluxes of N_2_O or CH_4_(mg m^−2^ h^−1^), V is volume (m^3^), M_0_ is the molecular weight of the gas, C_1_ and C_2_ are the concentration of previous (0 mins) and current (20 mins) gas concentrations inside the chamber (mol mol^−1^), T_1_ and T_2_ are temperature (Kelvin) recorded inside the chamber during current and previous samplings, and t_1_ andt_2_ are previous and current sampling times (h).

The cumulative emission of N_2_O and CH_4_ in kg ha^−1^ was estimated using the equation as follows (Yeboah et al., 2016):

(2) \begin{eqnarray*}M=\sum \left( {F}_{N+1}+{F}_{N} \right) \times 0.5\times \left( {t}_{N+1}-{t}_{N} \right) \times 24\times 1{0}^{-2}\end{eqnarray*}

where M is the N_2_O and CH_4_ cumulative emissions during the period of measurement (kg ha^−1^), F is N_2_O and CH_4_ emission (in mg m^−2^ h^−1^); and previous and current sampling emissions were N+1 and N respectively. The number of days from first sampling is represented by t.

### Biomass and grain yield

Biomass and grain yield was determined by cutting the plants using hand sickles to five cm height aboveground. The outer edges of about 0.5 m was discarded from each plot. Both yields were determined on a dry–weight basis by oven–drying the plant material at 105 °C for 45 min and then to constant weight at 85 °C (Yeboah et al., 2016).

### Statistical analyses

Statistical analyses were undertaken with the SPSS 22 (IBM Corporation, Chicago, IL, USA) with the treatment as the fixed effect and year as random effect. Tukey’s honestly significant was used to determine the differences between-treatments means. Significance differences were declared at probability level of 5%.

## Results

### Soil bulk density, saturated hydraulic conductivity and soil organic carbon

Soil samples taken during the study period showed significant differences in the bulk density depending on the type of treatment and the depth of sampling (Table 2). Bulk density increased with soil depth in many cases irrespective of treatment over the experimental period. Significant differences between treatments were minor in the upper layer in 2014, but significant treatment effect was recorded in the 5–10 cm soil depth as the experimental period progressed from 2014 to 2016 (Table 3). On average, the lowest bulk density (1.14 g cm^−3^) was recorded under biochar–amended soils, and the highest was observed under soils with carbon (1.21 g cm^−3^). The results obtained with the straw–amended soils showed a similar trend, except that differences were not significant at *p* < 0.05 in most cases. Saturated hydraulic conductivity (Ksat) was significantly (*p* < 0.05) affected by carbon, N fertilizer and year but there was no significant interaction between treatment factors (Table 2). Application of BN_100_ treatment enhanced mean saturated hydraulic conductivity by 23.7%, 24.3% and 20.4% relative to CN_0_, CN_50_ and SN_0_, respectively (Table 4). Carbon and year had significant interaction (*p* < 0.05) on soil organic carbon, except at the 10–30 cm soil depth (Table 2). Similarly, carbon and fertilizer-N also interactively affected soil organic C in all the soil depth evaluated. Application of fertilizer-N at the 50 and 100 kg ha^−1^ rate influenced SOC significantly (*p* < 0.05) under biochar treated soils, particularly in the depth of 0–5 cm (Table 5). However, N_100_ had greater effect compared to N_50_.

**Table 2:** Analysis of variance for carbon, nitrogen and year effects and their interaction.

Sources	Soil bulk density			Soil organic carbon						
	0–5	5–10	Ksat	0–5	5–10	10–30	N_2_O	CH_4_	Biomass yield	Grain yield
Carbon (C)	^**^	^*^	^*^	^*^	^*^	n.s.	^**^	n.s.	^**^	^**^
Nitrogen (N)	^**^	n.s.	^*^	^**^	^*^	n.s.	^**^	n.s.	^**^	^**^
Year (Y)	n.s.	^*^	^*^	^*^	n.s.	n.s.	^*^	^**^	^*^	^**^
C ×N	n.s.	n.s.	n.s.	n.s.	n.s.	n.s.	n.s.	^*^	^*^	n.s
C ×Y	n.s.	n.s.	n.s.	^**^	^**^	n.s.	n.s.	^**^	n.s	n.s
N ×Y	n.s.	n.s.	n.s.	n.s.	n.s.	n.s.	n.s.	n.s.	n.s	^**^

**Notes.**

*, ** Indicate significant difference at *P* < 0.05 and *P* < 0.01, respectively. n.s. indicate no significance difference at *P* < 0.05.

**Table 3:** Soil bulk density as affected by carbon addition sources.

Treatment		Soil BD (g cm^−3^)					
C source	Mineral N	0–5	5–10				10–30
		cm				
		Mean		2014	2015	2016	Mean	Mean
No carbon	N_0_	1.24a		1.32a	1.30ab	1.27a	1.29a	1.29a
	N_50_	1.17bc		1.24a	1.25abc	1.17bc	1.22abc	1.24a
	N_100_	1.20ab		1.20a	1.17cd	1.12c	1.16bc	1.27a
Biochar	N_0_	1.17bc		1.25a	1.25abc	1.24ab	1.25abc	1.27a
	N_50_	1.13cd		1.24a	1.16cd	1.14c	1.18bc	1.24a
	N_100_	1.15bcd		1.21a	1.18bcd	1.17bc	1.19bc	1.27a
Straw	N_0_	1.21ab		1.22a	1.32a	1.23ab	1.25ab	1.29a
	N_50_	1.11d		1.24a	1.08d	1.14c	1.16c	1.21a
	N_100_	1.14cd		1.28a	1.18cd	1.16bc	1.21abc	1.25a

**Notes.**

Values with different letters within a column are significantly different at *P* < 0.05.

**Table 4:** Saturated hydraulic conductivity as affected by carbon addition sources.

Treatment Saturated hydraulic conductivity (mm h^−1^)					
C source	Mineral N	2014	2015	2016	mean
No carbon	N_0_	62.64b	68.86b	62.95c	64.82c
	N_50_	65.77ab	64.06b	63.71c	64.51c
	N_100_	67.63ab	60.58b	75.45abc	67.89ab
Biochar	N_0_	71.93ab	62.88b	68.07bc	67.63ab
	N_50_	80.49a	70.94ab	78.98ab	76.80ab
	N_100_	78.78ab	78.99a	82.79a	80.19a
Straw	N_0_	68.66ab	65.65b	65.42c	66.58bc
	N_50_	76.07ab	66.02b	74.75abc	72.28ab
	N_100_	75.65ab	72.44ab	71.24abc	73.11ab

**Notes.**

Values with different letters within a column are significantly different at *P* < 0.05. *n* = 3.

**Table 5:** Soil organic carbon as affected by different treatments.

Treatment		Soil organic C (g kg^−1^)							
C source		N rate		0–10					10–30				
		2014		2015		2016	Mean	2014	2015		2016	Mean
No carbon	N_0_		9.64c		9.86c		10.43e		9.98d	9.29b	9.58b		9.48e	9.45c
	N_50_		10.18bc		9.92bc		11.54d		10.55cd	10.34ab	9.73b		10.71d	10.26bc
	N_100_		10.32bc		10.90bc		11.70d		10.97bcd	9.76ab	10.10b		11.05cd	10.30bc
Biochar	N_0_		11.82ab		10.28bc		14.91b		12.34b	10.45ab	10.27b		12.47b	11.06b
	N_50_		12.21ab		14.04a		16.01a		14.09a	11.59a	12.66a		14.41a	12.89a
	N_100_		12.42a		14.09a		16.26a		14.26a	11.04ab	13.75a		15.41a	13.40a
Straw	N_0_		9.71c		10.14bc		11.41d		10.42cd	9.58ab	10.12b		10.69d	10.13bc
	N_50_		10.70abc		10.64bc		13.77c		11.70bc	10.70ab	10.41b		11.54bcd	10.88b
	N_100_		11.08abc		11.19b		14.19bc		12.15b	10.92ab	10.99b		12.10bc	11.34b

**Notes.**

Values with different letters within a column are significantly different at *P* < 0.05. *n* = 3.

### Nitrous oxide emissions

**Figure 2:**
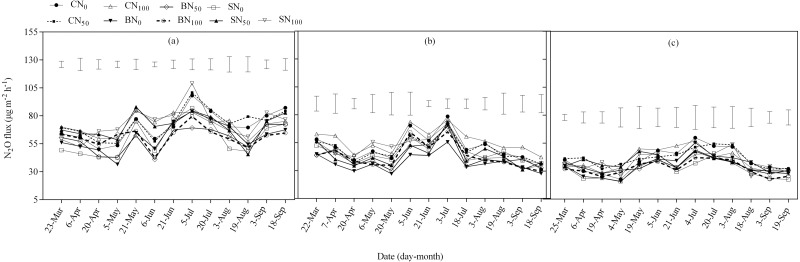
Seasonal N_2_O fluxes for spring wheat in 2014 (A), 2015 (B) and 2016 (C) as affected by carbon addition sources. The vertical bars represent the least significant difference (LSD) at *P* < 0.05 among treatments within a measurement date.

**Figure 3:**
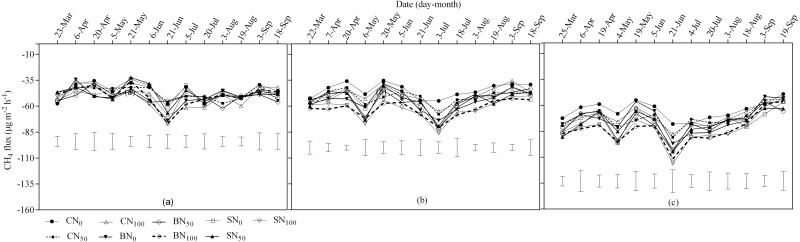
Seasonal CH_4_ fluxes for spring wheat in 2014 (A), 2015 (B) and 2016 (C) as affected by carbon addition sources. The vertical bars represent the least significant difference (LSD) at *P* < 0.05 among treatments within a measurement date.

All the treatments were sources of nitrous oxide (N_2_O) emission throughout the sampling period and the maximum observed N_2_O emissions occurred in early July in each year of this study (Fig. 2). These responses were consistent with recorded soil moisture and temperature data. Significant differences (*p* < 0.05) were found among treatments at certain periods of measurement (Fig. 3). For example, in 2014, the maximal N_2_O emission of BN_100_ was 79.5 µg m^−2^ h^−1^ and the minimal was 36.5 µg m^−2^ h^−1^; they were significantly lower than those for CN_50_ (100.7 µg m^−2^ h^−1^ for maximum and 55.8 µg m^−2^ h^−1^ for minimum) and CN_0_ (98.1 µg m^−2^ h^−1^ for maximum and 50.2 µg m^−2^ h^−1^ for minimum). At a lesser magnitude, SN_0_ and SN_50_ also produced significantly lower N_2_O emission compared to CN_0_ and CN_50_. During this period the lowest seasonal N_2_O emission was mostly recorded in the biochar treated soils and at a lesser magnitude in the straw treated soils.

There were no significant treatment interactions (*p* < 0.05) effect on cumulative N_2_O emission (Table 6), but treatment factors independently influenced cumulative N_2_O emission. The highest cumulative N_2_O emissions were consistently observed in the fertilized soils compared to the unfertilized soils, but differences were not always significant (Table 6). Application of BN_0_, BN_50_ and BN_100_ significantly decreased cumulative N_2_O emission by 48.42%, 37.12% and 35.80% on average compared to CN_100_, respectively (Table 4). The mean cumulative N_2_O emission of biochar was averaged at 1.83 kg ha^−1^ representing significant decrease of 10.93% and 38.61% compared to straw treated soils (2.03 kg ha^−1^) and soils without carbon treatment (2.42 kg ha^−1^). Straw treated soils had non–significant cumulative N_2_O decrease of 0.39 kg ha^−1^, or 19.40% less compared to no carbon soils.

**Table 6:** Cumulative N_2_O emissions of spring wheat as affected by carbon addition sources.

Treatment		N_2_O (kg ha^−1^)			
C source	N rates	2014			2015		2016		Mean	
No carbon	N_0_	3.10a			2.09ab		2.00ab		2.40ab	
	N_50_	3.17a			2.00ab		1.75bc		2.31abc	
	N_100_	3.21a			2.37a		2.11a		2.56a	
Biochar	N_0_	2.37b			1.50c		1.32c		1.73c	
	N_50_	2.47b			1.73bc		1.41c		1.87bc	
	N_100_	2.48b			1.77bc		1.42bc		1.89bc	
Straw	N_0_	2.43b			1.87bc		1.24c		1.85bc	
	N_50_	2.83ab			1.78bc		1.54abc		2.05abc	
	N_100_	2.99a			1.98ab		1.61abc		2.19abc	

**Notes.**

Values with different letters within a column are significantly different at *p* < 0.05.

### Methane emissions

All the treatments had similar trends of seasonal CH_4_ dynamics and were net carbon sinks over the three study years (Fig. 3). The minimum CH_4_ consumption was recorded in April 2014 and 2015, and in September 2016. In the present study, a single peak was observed in June 2014, whiles double peaks were observed in May and July 2015 and 2016. During this period, the greatest seasonal CH_4_ consumption of –79.94, –81.07 and –111.59 µg m^−2^ h^−1^ in 2014, 2015 and 2016 respectively were observed in BN_100_ soils; it was 38.14%, 47.37%, 43.05% more compared to CN_0_ (–57.87, –55.01 and –78.01 µg m^−2^ h^−1^). At a lesser extent, the maximum seasonal CH_4_ consumption in SN_50_ and SN_100_ soils were significantly higher (*p* < 0.05) compared to the CN_0_ and CN_50_ soils. The results were clear that, the greater seasonal CH_4_ consumption occurred with the higher N fertilizer soils and the greatest CH_4_ uptake generally occurred in the biochar treated soils, followed by the straw treated soils and the least were observed in the no carbon soils

Year individually had a significant effect (*p* < 0.05) on cumulative CH_4_ emission (Table 7), and interaction between carbon and year significantly affected cumulative CH_4_ emission. The results of cumulative CH_4_ emission showed that increasing N fertilizer rates generally enhanced CH_4_ consumption in all treatments. The use of BN_100_ boosted cumulative CH_4_ uptake in 2014 (by 21.9% and 18.2%), 2015 (by 83.6% and 59.1%) and 2016 (by 30.5% and 18.4%) compared to CN_0_ and CN_50_, respectively. Increasing the fertilizer rate from N_50_ to N_100_ resulted in significantly higher cumulative CH_4_ consumption (*p* < 0.05) on straw treated soils in 2014 relative to N_0_ on soils without carbon; the increase was 16.8%. In 2015, application of SN_100_ increased cumulative CH_4_ sink by 41.0%, 73.0%, 22.8% and 26.8% compared with CN_0_, CN_50_ and CN_100_, respectively. The mean cumulative CH_4_ consumption was greatest in biochar treated plots (−2.8 kg ha^−1^), followed by straw treated soils (−2.6 kg ha^−1^) and the least in no carbon soils (−2.3 kg ha^−1^).

**Table 7:** Cumulative CH_4_ emissions of spring wheat as affected by different treatment.

Treatment		CH _4_(kg ha^−1^)			
C source	N rates	2014	2015	2016	Mean
No carbon	N_0_	–1.80a	–1.79a		–2.83a			–2.14a	
	N_50_	–1.85ab	–2.07a		–3.12ab			–2.35ab	
	N_100_	–2.08bc	–2.00a		–3.13abc			–2.40abc	
Biochar	N_0_	–2.09bc	–3.14c		–3.31abc			–2.85bc	
	N_50_	–2.13bc	–2.23ab		–3.38abc			–2.58abc	
	N_100_	–2.19c	–3.29c		–3.70c			–3.06c	
Straw	N_0_	–1.91abc	–2.16ab		–3.19abc			–2.42abc	
	N_50_	–1.96abc	–2.21ab		–3.32abc			–2.50abc	
	N_100_	–2.10bc	–2.54b		–3.61bc			–2.75abc	

**Notes.**

Values with different letters within a column are significantly different at *P* < 0.05.

### Biomass and grain yield

There was significant interaction effects between carbon and nitrogen, and nitrogen and year on biomass yield at *p* < 0.05 (Table 2). In addition, carbon, nitrogen and year individually had significant effect on biomass yield. Application of N_100_ treatments on biochar treated soils (BN_100_) increased biomass yield by 39.05% in 2014, 37.31% in 2015 and 30.02% in 2016 on average compared to soils without carbon (Table 8). Similarly, BN_100_ significantly increased biomass yield in 2014 (by 35.06% and 26.43%), 2015 (by 40.04% and 23.11%) and 2016 (by 21.86% and 13.45%) compared to SN_0_ and SN_50_ sites, respectively. Application of SN_100_ also caused significant increases in biomass yield compared to no carbon soils, an average increase of 32.09%, 29.32% and 32.56% were recorded in 2014, 2015 and 2016 respectively. The grain yield under N_100_ fertilization was significantly increased (*p* < 0.05) by 35.87%, 29.45% and 13.34% under no carbon soils; 33.64%, 37.02% and 39.16% under biochar soils, and 31.89%, 32.35% and 24.08% under biomass treated soils in 2014, 2015 and 2016, respectively, compared to their corresponding N_0_ soils (Table 9).

**Table 8:** Biomass yield of spring wheat as affected by different treatment.

Treatment		Biomass yield (kg ha^−1^)			
C source	N rates	2014	2015	2016	Mean
No carbon	N_0_		2776d			3030d				2455d	2754c			
	N_50_		3102c			3358bcd				3022c	3161bc			
	N_100_		3399bc			3739b				3267bc	3468b			
Biochar	N_0_		3295bc			3530bc				3147bc	3324b			
	N_50_		3489b			3767b				3331bc	3529b			
	N_100_		4291a			4630a				3788a	4236a			
Straw	N_0_		3170bc			3312cd				3118bc	3200bc			
	N_50_		3403bc			3765b				3365b	3511b			
	N_100_		4082a			4345a				3633a	4020b			

**Notes.**

Values with different letters within a column are significantly different at *P* < 0.05.

**Table 9:** Grain yield of spring wheat as affected by different treatments.

Treatment		Grain yield (kg ha^−1^)			
C source		N rates		2014		2015			2016			Mean
No carbon	N_0_		1305d		1500d			1009d			1271d	
	N_50_		1538cd		1896bc			1043cd			1492bcd	
	N_100_		1770abc		1927bc			1144cd			1614cd	
Biochar	N_0_		1603bcd		1789cd			1124cd			1505bcd	
	N_50_		1905abc		2133b			1233bc			1757abc	
	N_100_		2139a		2456a			1567a			2054a	
Straw	N_0_		1502cd		1658cd			1111cd			1424cd	
	N_50_		1852abc		1944bc			1182cd			1659bc	
	N_100_		1975ab		2180ab			1380ab			1845ab	

**Notes.**

Values with different letters within a column are significantly different at *P* < 0.05.

## Discussion

The lowest cumulative N_2_O emission was recorded in the biochar treated soils and at a lesser magnitude in the straw treated soils, whereas the highest N_2_O emission was observed in the no carbon treated soils. In both cases, the highest rate of N fertilizer recorded the greatest N_2_O emission. In contrast, Chatskikh (2007) and Kammann et al. (2012) reported that N_2_O fluxes were significantly increased by addition of biochar, particularly when added with mineral N-fertilizer. It has been shown that the type and rate of fertilizer have an important impact on N_2_O emissions (Bouwman, Boumans & Batjes, 2002). Some studies have reported that use of crop straw combined with mineral nitrogen fertilizer enhances soil quality while reducing N_2_O emissions (Xu, Han & Ru, 2019; Sainju, 2016). Crop straw return commonly aims at improving soil carbon and nitrogen cycling (Xu, Han & Ru, 2019; Meng et al., 2017), thought it can also be a source of trace gas emissions (Cha et al., 2016). Nitrogen fertilization has the greatest potential to increase N_2_O emissions because mineral N controls both nitrification and denitrification. Other studies (Zhang et al., 2011) have shown that biochar combined with N-fertilizer can significantly reduce N_2_O emissions. One mechanism that may explain lower (cumulative) N_2_O fluxes from biochar + N-fertilizer-amended soils is the fact that relatively low C soils treated with N-fertilizer and biochar may retain relatively higher amounts of mineral N than soils untreated with N-fertilizer (Zhang et al., 2011). Nitrogen thereby retained provides a source of available N for plant uptake, which reduces N availability for microbes involved in denitrification processes. Since biochar has significant impact on soil environment and affects many soil parameters such as the availability of substrates (Van Zwieten et al., 2009), it is very likely that biochar will have significant effects on the production of N_2_O. Their results is confirmed by the increased plant N uptake in this study (Table S4). Singh et al. (2010) reported that biochar can also reduce the N availability to microorganisms by absorption. In this study, improved soil porosity could also explain the decreased N_2_O emission recorded when biochar was applied with N fertilizer. Soil aeration and improved porosity inhibit denitrification. Nitrogen dynamics are affected by changes in soil aeration, pH and the C/N ratio of the material incorporated into the soil. Biochar may suppress N_2_O production from denitrification by increasing the air content of the soil or by absorbing water from the soil, thus improving aeration of the soil (Yanai, Toyota & Okazaki, 2007). Karhu et al. (2011) shared similar view and observed that biochar amendment modifies soil physical properties such as reducing soil bulk density or increasing water holding capacity (Karhu et al., 2011), thereby increasing soil aeration. This may lead to lower soil N_2_O emissions, as soil aeration influences both nitrifier and denitrifier activity. Soils, which are not affected by compaction often exhibit adequate porosity and therefore the risk of denitrification is lower compared with soils that have impaired infiltration or internal drainage (Antille, 2018). In this study, lower N_2_O emissions were also observed on the straw treated plots, although the effects were lesser relative to the biochar treated soils. The lower N_2_O emission under straw treated soils could be attributed to the accumulation of organic matter on the soil surface that led to reduced bulk density and thus improved soil aeration.

Reductions in CH_4_ emission were observed in biochar–amended soils and to a lesser extent on straw amended soils compared to their controls. Literature evidence indicated that biochar input to soil can potentially reduce CH_4_ emissions (Yeboah et al., 2018). In contrast, Xie et al. (2021) showed that charcoal input into soil may increase soil methane fluxes. The mechanisms underlying changes in soil CH_4_ emissions following biochar amendment are unclear (Lehmann et al., 2011). The greater uptake of CH_4_ may be attributed to the protected environment created for the CH_4_ oxidizers and improved soil porosity. In this study, the greater uptake of methane in the soils with carbon amendment, particularly biochar amended soils with N fertilizer may be attributed to the favorable environment created for the CH_4_ oxidizers. The aerobic, well drained soils can be a sink for CH_4_ due to the possible high rate of CH_4_ diffusion and ensuing oxidation by methanotrophs. Combined application of biochar and inorganic N-fertilizer in this study improved soil physical properties (reduction in soil bulk density and increased soil saturated hydraulic conductivity) *.* Such improved soil structural conditions are known to protect the ecological niche for methanotrophic bacteria, influence the gaseous diffusivity, and affect the rate of supply of atmospheric CH_4_ (Hütsch, 1998; Serrano-Silva et al., 2014; Ma et al., 2016). Aerobic, well–drained soils behave as a sink for CH_4_ due to the high rates of CH_4_ diffusion and subsequent oxidation by methanotrophs (Serrano-Silva et al., 2014). However, these results do not appear to support the conclusions of Laird (2008) on the reduction observed in methane emissions from field plots, which was deduced as an increased CH_4_ oxidation activity. Other studies have reported significant increase in CH_4_ emissions following biochar or biomass application (*e.g.*, Wang et al., 2012). The authors explained that, the increased availability of labile C substrates following biochar or biomass addition stimulates the activities of methanogenic bacteria which may account for increased CH_4_ emissions. However, this could be a short-term effect since labile carbon fraction in the materials could be mineralized rapidly (Wang et al., 2012).

The results of this study indicate that when biochar was applied together with fertilizer N, both biomass and grain yield of spring wheat increased. This finding shows the potential of biochar applied together with fertilizer N to improve nutrient use efficiency in spring wheat in semiarid environment (Solaiman et al., 2010). Diverse reasons have been given to the positive effect of biochar applied in combination with fertilizer N on crop yield. Bruun et al. (2011) reported that combined application of biochar and N fertilizer has the potential to improve soil properties and could therefore be responsible for the effect observed. Similarly, both Borchard et al. (2012) and Tammeorg et al. (2014) attribute increased crop productivity when biochar is applied together with N fertilizer to improve nutrient availability. In the current study, increased yield may be attributed to increased nutrient availability and improved soil physical and chemical properties (soil bulk density, saturated hydraulic conductivity and soil organic carbon), as reported in earlier work (Zhang et al., 2010). These results imply that, when biochar and inorganic fertilizers are applied together, an increased nutrient supply to plants may be the most important factor in increasing crop yields. The higher biomass and grain yield obtained on the carbon amended soils compared to the soils without carbon in this study is attributed to the fact that in drier soils,crop residues provide a better soil environment by reducing temperature, conserving water, and improving soil quality resulting in better yield (Zou et al., 2016). Positive effects of biochar combined with N fertilizer on increasing SOC and hydraulic conductivity as well as decreasing soil bulk density was observed in this study. Therefore, this study evidenced a positive effect of biochar amendment on soil quality and spring wheat yield consistent over three consecutive years. Furthermore, the lowest yield recorded on the no carbon soils throughout this study may be related to the removal of all the aboveground biomass at the end of the cropping season. Zhang, Yang & Wu (2008) showed that field practices with low carbon inputs to arable soils as crop biomass removal and manure abandonment deplete soil organic carbon and reduce crop productivity. Therefore, when biochar was applied and crop residues retained, it had immediate effect and the beneficial influence on biomass and grain yields were obtained.

## Conclusions

Application of crop residue amendments combined with nitrogen fertilizer has been increasingly recommended as an effective management practice for mitigating greenhouse gas emissions while enhancing soil fertility, thereby increasing crop production. In this paper, we have shown that application of carbon amendment, especially biochar combined with N fertilizer in wheat grown under rain fed conditions in a semi-arid environment reduced nitrous oxide and methane emissions whilst increasing biomass and grain yield. This study confirmed our hypothesis that increased C inputs would increase the soils ability to reduce N_2_O and CH_4_ soil emissions whiles increasing biomass and grain yield. The main conclusions derived from this work are: application of biochar + N-fertilizer (BN_100_) or straw + N-fertilizer (SN_100_) increased saturated hydraulic conductivity to significantly greater extent than the other treatments tested. This translated into higher biomass production and therefore grain yield in those treatments. These results indicate the dependency of crop yield on soil quality and imply that crop productivity could be increased without resource degradation when biochar is applied combined with N-fertilizer. Application of biochar + N-fertilizer showed relatively lower N_2_O emissions, including increased uptake of CH_4_, but the effect of BN_100_ was consistently greater. The findings of this study suggest that biochar applied together with N-fertilizer can concurrently improve soil physical and chemical properties as well as biomass and grain yield while reducing the effect of agricultural activities on the environment. Based on this results, the potential exist for developing crop and soil management interventions around biochar applied together with fertilizer N in semiarid environments. Further studies that focus on N_2_O and CH_4_ measurements after every rainfall, tillage and fertilization events are required for better recommendations.

##  Supplemental Information

10.7717/peerj.11937/supp-1 Supplemental Information 1 Raw data set of N2O and CH4 emission N =3

10.7717/peerj.11937/supp-2 Supplemental Information 2 Raw crop yield data n =3

10.7717/peerj.11937/supp-3 Supplemental Information 3 Raw soil data n =3

10.7717/peerj.11937/supp-4 Supplemental Information 4 Total nitrogen of spring wheat plant as affected by carbon addition sources

## Additional Information and Declarations

### Data Availability

The following information was supplied regarding data availability: The raw data for N_2_O and CH_4_, grain and biomass yield, soil data and Plant N are available in the Supplemental Files.
