# Supplementary material for: Nitrous oxide, methane emissions and grain yield in rainfed wheat grown under nitrogen enriched biochar and straw in a semiarid environment
Source: PeerJ. 2021 Aug 19;9:e11937. doi: 10.7717/peerj.11937 (PMC8380423; doi:10.7717/peerj.11937)
Supplement: Supplemental Information 4 [file peerj-09-11937-s004.docx]

| Treatment | | | | | |
| --- | --- | --- | --- | --- | --- |
| C source | Mineral N | Plant N (g kg^–1^) | |  |  |
|  |  | 2014 | 2015 | 2016 | Mean |
| No carbon | N_0_ | 5.01cd | 5.33bc | 6.07b | 5.47b |
|  | N_50_ | 5.05cd | 5.06c | 6.31ab | 5.47b |
|  | N_100_ | 5.40bc | 5.28bc | 6.79a | 5.82ab |
|  |  |  |  |  |  |
| Biochar | N_0_ | 5.04cd | 5.06c | 6.09b | 5.40b |
|  | N_50_ | 5.34bcd | 5.37bc | 6.59ab | 5.77ab |
|  | N_100_ | 5.53b | 5.96a | 6.95a | 6.15a |
|  |  |  |  |  |  |
| Straw | N_0_ | 4.98d | 5.29bc | 6.00b | 5.42b |
|  | N_50_ | 5.34bcd | 5.55ab | 6.51ab | 5.80ab |
|  | N_100_ | 6.38a | 5.64ab | 6.86a | 6.29a |

Values with different letters within a column are significantly different at *P*<0.05. n=3
